# Effects of a novel non-pharmacological intervention based on respiratory biofeedback, neurofeedback and median nerve stimulation to treat children with ADHD

**DOI:** 10.3389/fnhum.2025.1478501

**Published:** 2025-02-07

**Authors:** Eduardo Santamaría-Vázquez, Anayali Estudillo-Guerra, Lna Ali, Diana Martinez, Roberto Hornero, Leon Morales-Quezada

**Affiliations:** ^1^Biomedical Engineering Group, University of Valladolid, Valladolid, Spain; ^2^Centro de Investigación Biomédica en Red en Bioingeniería, Biomateriales y Nanomedicina, Madrid, Spain; ^3^Department of Physical Medicine and Rehabilitation, Spaulding Rehabilitation Hospital, Harvard Medical School, Boston, MA, United States; ^4^Boston Neurodynamics, Boston, MA, United States

**Keywords:** attention and hyperactivity deficit disorder (ADHD), median nerve stimulation (MNS), neurofeedback (NFB), respiratory biofeedback, electroencephaloagraphy (EEG)

## Abstract

**Introduction:**

Attention deficit hyperactivity disorder (ADHD) is a neurodevelopmental condition that affects cognitive, academic, behavioral, emotional, and social functioning, primarily in children. Despite its high prevalence, current pharmacological treatments are not effective in 30% of cases and show poor long-term adherence. Non-pharmacological interventions can complement medication-based treatments to improve results. Among these therapies, neurofeedback (NFB) and respiratory biofeedback (R-BFB) have shown promise in treating ADHD symptoms. Moreover, median nerve stimulation (MNS) can help to enhance the efficacy of these treatments, but it has never been explored in this context. This study aimed to: (1) investigate the effectiveness of a combined R-BFB and NFB intervention to treat ADHD, and (2) explore the potential benefits of MNS in enhancing the proposed intervention.

**Methods:**

Sixty children with ADHD participated in the study, divided into two experimental groups. The active group received *verum* MNS, and the sham group received sham MNS. Both groups performed the NFB/R-BFB treatment. Clinical assessments (i.e., Conner's parent rating scale) and electroencephalography (EEG) measurements were taken before the intervention, immediately after treatment, and one month later.

**Results:**

The results showed that the combined therapy significantly improved behavioral problems, anxiety, hyperactivity, and impulsivity-hyperactivity. Moreover, MNS enhanced the positive effects of the intervention, as the active group achieved higher improvement compared to the sham group. EEG analysis revealed significant changes in spontaneous brain activity, with an increase in frontal theta power (*p* = 0.0125) associated with reduced anxiety, which might explain the clinical outcomes. These changes were maintained 1 month after the intervention (*p* = 0.0325). Correlations between EEG changes and clinical outcomes were observed, suggesting a potential relationship between neurophysiological markers and ADHD symptoms measured by standardized scales.

**Discussion:**

The study findings suggest that the proposed R-BFB/NFB intervention may be an effective non-pharmacological therapy for ADHD, with the additional application of MNS potentially enhancing its effects.

## 1 Introduction

Attention deficit hyperactivity disorder (ADHD) is a neurodevelopmental condition that usually manifests in childhood with hyperactivity, impulsivity, and/or inattention symptoms that affect cognitive, academic, behavioral, emotional, and social functioning (Association, 2013). ADHD is common in children, with an estimated prevalence between 3% and 10%, depending on the diagnostic criteria and the analyzed population (Danielson et al., 2018; Thomas et al., 2015). Boys are more likely to be diagnosed with ADHD than girls (12.9% compared to 5.6%) (Danielson et al., 2018). In addition, many children with ADHD also have other mental health disorders, with 6 out of 10 showing behavior problems, or anxiety (Danielson et al., 2018).

The diagnostic criteria for ADHD have evolved over time, but the assessment and evaluation tools have not changed much. Clinical diagnosis remains the gold standard for ADHD, including a comprehensive history taking of prenatal, perinatal, and family history; school performance; environmental factors; and a detailed physical examination (Wolraich et al., 2019a; Faraone et al., 2021). The objective assessments currently available for ADHD are of limited use for diagnosis; neuropsychological tests have a low strength of evidence (Kemper et al., 2018); and EEG and neuroimaging evidence remains insufficient (Kemper et al., 2018). Currently, the most widely accepted criteria for ADHD diagnosis is outlined in the Diagnostic and Statistical Manual of Mental Disorders Fifth Edition (DSM 5 TR) (Association, 2013). While it is highly unlikely that diagnostic biomarkers will replace clinical assessment, they may eventually support clinical decision-making. Numerous studies have revealed structural brain disparities between children with and without ADHD, such as asymmetry of the caudate nucleus, smaller cerebral and cerebellar volumes, reduced posterior corpus callosum regions, and increased gray matter in certain cortices (Sáenz et al., 2019; Giedd et al., 2006; Jacobson et al., 2018; Sowell et al., 2003). However, a recent meta-analysis that includes 96 studies and 1914 participants found a lack of consistency in regional differences among children with ADHD, possibly due to variations in clinical populations, experimental designs, preprocessing methods, and statistical procedures. In terms of structural connectivity studies using diffusion-weighted imaging (DWI), individuals with ADHD exhibited reduced fractional anisotropy, primarily in frontostriatal pathways, the cingulum, and the cingulate cortex (Parlatini et al., 2023). Functional MRI (fMRI) studies have shown global basal ganglia activation and reduced anterior frontal lobe activation, (Bush et al., 1999; Zang et al., 2005; Hart et al., 2013; McCarthy et al., 2014; Cortese et al., 2012) consistent with EEG findings, including aberrant topographic distribution for theta, alpha, and beta spectral power, or increased theta activity over prefrontal-central structures during cognitive testing (Snyder and Hall, 2006; Lenartowicz and Loo, 2014). These findings support the hypothesis of atypical cortical frontal-striatal-thalamocortical (CSTC) function in ADHD, characterized by cortical hyperarousal and a lack of inhibition of irrelevant sensory input. However, factors such as subject-specific developmental differences and the use of simplified spectral metrics based on fixed EEG bands limit the generalizability of these findings (Loh et al., 2022). While neuroimaging and neurophysiological techniques hold promise for studying ADHD pathophysiology and complementing neuropsychological evaluations, further research is warranted.

Medication and behavioral techniques based on behavior modification have been found to be effective treatments for children and adolescents with ADHD (Farmer et al., 2002). Numerous studies have demonstrated the efficacy of medication for treating the core symptoms of ADHD (Jensen, 1999). However, about 30% of children do not respond to these treatments or experience adverse side effects (Wolraich et al., 2019b). Long-term adherence to a medication regimen is poor, with most estimates suggesting that fewer than 50% of children with ADHD maintain prescribed dosages over a period of 6 months (Hoagwood et al., 2000). In view of these results, non-pharmacological alternatives to treat ADHD symptoms hold great promise, including different types of biofeedback (BFB). These techniques take advantage of the findings achieved through brain activity biomarkers (e.g., fRMRI, EEG) to propose novel treatment approaches. Concretely, Neurofeedback (NFB) is a specific type of BFB that enables individuals to gain control over their own brain activity (Enriquez-Geppert et al., 2017). In the NFB paradigm, a brain-computer interface (BCI) registers the EEG of the patient and calculates different metrics from this signal. These metrics are converted into real-time visual and auditory feedback, which is then presented to patients. This process empowers individuals to engage in self-regulation, allowing them to modulate the activity of specific brain regions linked to behaviors or symptoms in a personalized manner (Enriquez-Geppert et al., 2017). It is hypothesized that NFB promotes neuroplasticity by modulating synaptic long-term potentiation (LTP) or long-term depression (LTD) during the associative relationship between the EEG-derived activity and the visual and auditory stimulus presented to the subject (Gurevitch et al., 2024). Nevertheless, the efficacy of NFB treatments for ADHD remains a topic of debate. While numerous studies have reported promising results using various NFB protocols, the findings are not universally consistent. For instance, sensorimotor rhythm (SMR) NFB training targeting the sensorimotor area, which is functionally linked to behavioral inhibition, has shown notable reductions in ADHD symptoms among children (Krepel et al., 2020; Wang et al., 2022). Moreover, Doren et al. (2019) conducted a systematic review and meta-analysis, concluding that NFB represents a viable non-pharmacological treatment option for ADHD, with evidence suggesting that its therapeutic effects persist even after the intervention is completed and withdrawn. However, the study by the Group (2023) reported contrasting results, attributing improvements to non-specific effects. This interpretation is further supported by a recent meta-analysis by Westwood et al. (2024), which highlighted limitations in the generalizability of positive outcomes, particularly in blinded studies. Despite these inconsistencies, the cited studies emphasize the need for further investigation to determine the specific effects, if any, of NFB in this context. In particular, Westwood et al. (2024) advocate for further research on standardized NFB protocols, such as SMR training, which demonstrated statistically significant but modest improvements in ADHD symptoms in blinded studies. Other biofeedback interventions that proved useful for ADHD treatment are respiratory BFB (R-BFB), heart rate variability BFB (HRV-BFB), or temperature BFB (Schoenberg and David, 2014). Specially, R-BFB aims to retrain respiratory patterns to achieve effortless diaphragmatic breathing by using breathing sensors and an interface that allows the participant to pace breathing for the purpose of achieving a relaxation response (Schoenberg and David, 2014). All BFB techniques, including NFB and R-BFB, use the principles of operant conditioning to promote associative learning and self-regulation, with the goal of ameliorating ADHD symptoms (Schoenberg and David, 2014). Hence, the use of R-BFB might reduce anxiety, stress, and depressive symptoms associated with ADHD, while also facilitating performance during NFB training. Thus, the combined use of R-BFB and NFB may lead to a more effective treatment response and improvement across clinical outcomes.

Recent advances and growing evidence supporting the safety and efficacy of non-invasive neuromodulatory techniques in adults have facilitated the study of neuromodulation applications in children and adolescents. Noninvasive brain stimulation (NIBS) techniques, such as transcranial direct current stimulation (tDCS) and transcranial magnetic stimulation (TMS), have been considered in children with depression, autism spectrum disorder, ADHD, and other neuropsychiatric disorders (Camsari et al., 2018). However, changes in neural maturational states secondary to the stimulation must be considered when applying tDCS or TMS. An alternative to NIBS is peripheral noninvasive stimulation, where principles of bottom-up modulation can be used to promote learning processes (Carvalho et al., 2018). Among the peripheral targets for electrical stimulation is the median nerve. During the median nerve stimulation (MNS) at the wrist level, the electrical impulses follow the median nerve pathway reaching the ascending reticular formation in the brain stem, increasing the excitability in this area and modulating the sensorimotor thalamocortical pathways (M1 and S1 cortices), as well as the insula and other cortical structures (Ferretti et al., 2007). In individuals with ADHD, there is a documented link between deficits in executive control and motor inhibition (Barkley and Poillion, 1994). Research has consistently reported reduced activation in fronto-striatal and frontoparietal circuits during tasks requiring inhibition in this population (Hart et al., 2013; Cortese et al., 2012). When performing simple motor tapping, subjects with ADHD have shown decreased activation in the primary motor cortex relative to controls (Mostofsky et al., 2006). Additionally, reduced cortical inhibition has been correlated with deficits in motor performance (Gilbert et al., 2011). Therefore, we hypothesized that MNS could facilitate adequate behavioral responses by modulating thalamocortical inhibitory inputs. These circuits involve the sensorimotor system (M1/S1 cortex), acting as a primer for supplementary co-activation of distant networks when individuals are exposed to cognitive or physical tasks. Additionally, when targeted training is introduced, MNS appears to augment the effects of such activities by promoting processes linked to neuroplasticity (Houlgreave et al., 2022). Overall, following this rationale, MNS could serve as an adjunctive technique to facilitate inhibitory circuits and improve attention function in ADHD patients. Potentially helping modulate attention and filter interfering stimuli and therefore improve executive functions.

Therefore, MNS represents an affordable and noninvasive technique that has the potential to induce neurolasticity within somatosensory networks via the spinothalamic tract (Carvalho et al., 2018; Backes et al., 2000). MNS has demonstrated successfull applications in various neuropsychiatric disorders, including Tourette syndrome and anxiety (Maiquez et al., 2020). Given its characteristics, MNS also holds promise as an effective treatment option for ADHD. However, this technique has yet to be investigated in this condition. We hypothesize that MNS could enhance NFB and R-BFB training protocols, thereby increasing the effectiveness of these therapies in alleviating symptoms related to pediatric ADHD. Nevertheless, this multimodal intervention has not been tested in real clinical settings. The primary goal of this study is twofold: (1) to assess the efficacy of a combined therapy utilizing R-BFB and NFB for pediatric ADHD treatment; and (2) to explore whether MNS can enhance these interventions by comparing sham and real stimulation within a group design. To achieve these aims, we implemented the proposed protocol in 60 children with ADHD, evaluating both clinical and neurophysiological changes associated with these interventions.

## 2 Materials and methods

### 2.1 Participants

Study subjects were recruited from local pediatric clinics and referrals from pediatric centers at the Neuromodulation Center NEOCEMOD in Aguascalientes City, Mexico. A total of 60 participants were enrolled in the study. The inclusion criteria were: (1) ADHD diagnosis performed by a board-certified clinician according to the American Psychiatric Association's Diagnostic and Statistical Manual, Fifth edition (DSM-5) (Association, 2013) and the International Classification of Diseases, Tenth Revision (ICD-10) (Organization, 1992); (2) between 8 to 18 years of age; and (3) on stable medication doses for at least 3 months previous to enrollment. The exclusion criteria included: (1) comorbidity with severe neurological or psychiatric disorder; (2) comorbidity with uncontrolled chronic medical diseases such as diabetes, cardiopathies, or renal failure; (3) any other medical condition that in the view of the investigator could affect the participation of the patient.

### 2.2 Study protocol

**Figure 1:**
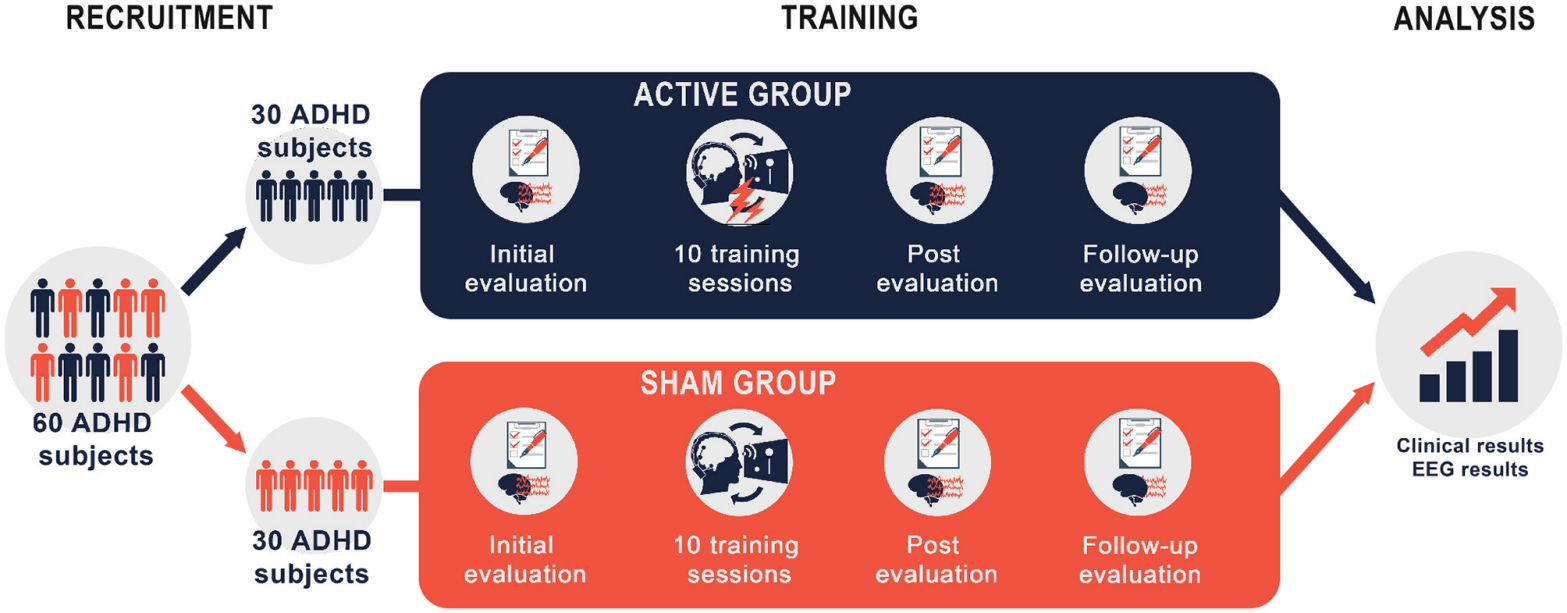
Schematic representation of the study protocol.

This was an exploratory randomized, double-blinded, sham-controlled, two-arm parallel-group clinical trial. The study was approved by the Bioethics and Research Committee (C.No.DG. UAA-BEC 002/17) from Universidad Autonoma de Aguascalientes (Aguascalientes, Mexico). Written informed consent from each participant's parent or legal guardian was obtained; participants were randomly assigned to the intervention groups using a computerized random number generator. Both groups underwent ten training sessions of 30 min each. Participants were evaluated at three-time points: pre-intervention, post-intervention, and 1-month follow-up. Compliance with the study's inclusion criteria was verified at each evaluation session. Particularly, changes in participants' pharmacological treatments were monitored though medication diaries. Figure 1 shows a schematic representation of the intervention protocol and the temporal distribution of the sessions. The data is available upon reasonable request. Requests to access the datasets should be directed to eduardo.santamaría.vazquez@uva.es.

### 2.3 Evaluation sessions

We assessed ADHD symptoms severity using the revised Conners' Parent Rating Scale (CPRS-R). The CPRS-R is a comprehensive tool used to assess behavioral, emotional, and academic issues in children. The CPRS-R is commonly used to help diagnose ADHD and other related disorders. The scale consists of a series of questions that parents answer based on their observations of their child's behavior. The CPRS-R includes multiple subscales such as Oppositional Behavior, Cognitive Problems/Inattention, Hyperactivity, Anxious-Shy, Perfectionism, Social Problems, and Psychosomatic issues. Each item on the scale is rated on a 4-point Likert scale: 0 = Not at all true (never, seldom); 1 = Just a little true (occasionally); 2 = Pretty much true (often, quite a bit); 3 = Very much true (very often, very frequent). The minimum score for each item is 0 while the maximum score for each item is 3. The total score is calculated by summing the ratings for each item, with higher scores indicating more severe behavioral or emotional problems. Then, these raw scores are converted to T-scores using normative data, which standardizes the results based on the child's age and gender. T-scores have a mean score of 50 with a standard deviation of 10, facilitating comparison against a representative population (Conners et al., 1998).

Awake EEG recordings were recorded at rest in closed-eyes condition, each recording lasting 5 minutes. EEGs were recorded according to the American Clinical Neurophysiology Society recommendations using the 10/20 International System (Acharya et al., 2016), at a sample rate of 500 Hz, amplified and filtered using a bandpass of 0.3–50 Hz using the EEG-amplifier Neuroamp II (BEE medic, Switzerland). The EEG assessment allows for analyzing the spontaneous cortical activity of the brain. This activity provides valuable information about the functional state of endogenous brain oscillations and has been widely used to assess ADHD (Lenartowicz and Loo, 2014). Multiple biomarkers have been proposed to study this pathology and may provide objective measurements to support the clinical outcomes of the study (Lenartowicz and Loo, 2014).

### 2.4 Training sessions

**Figure 2:**
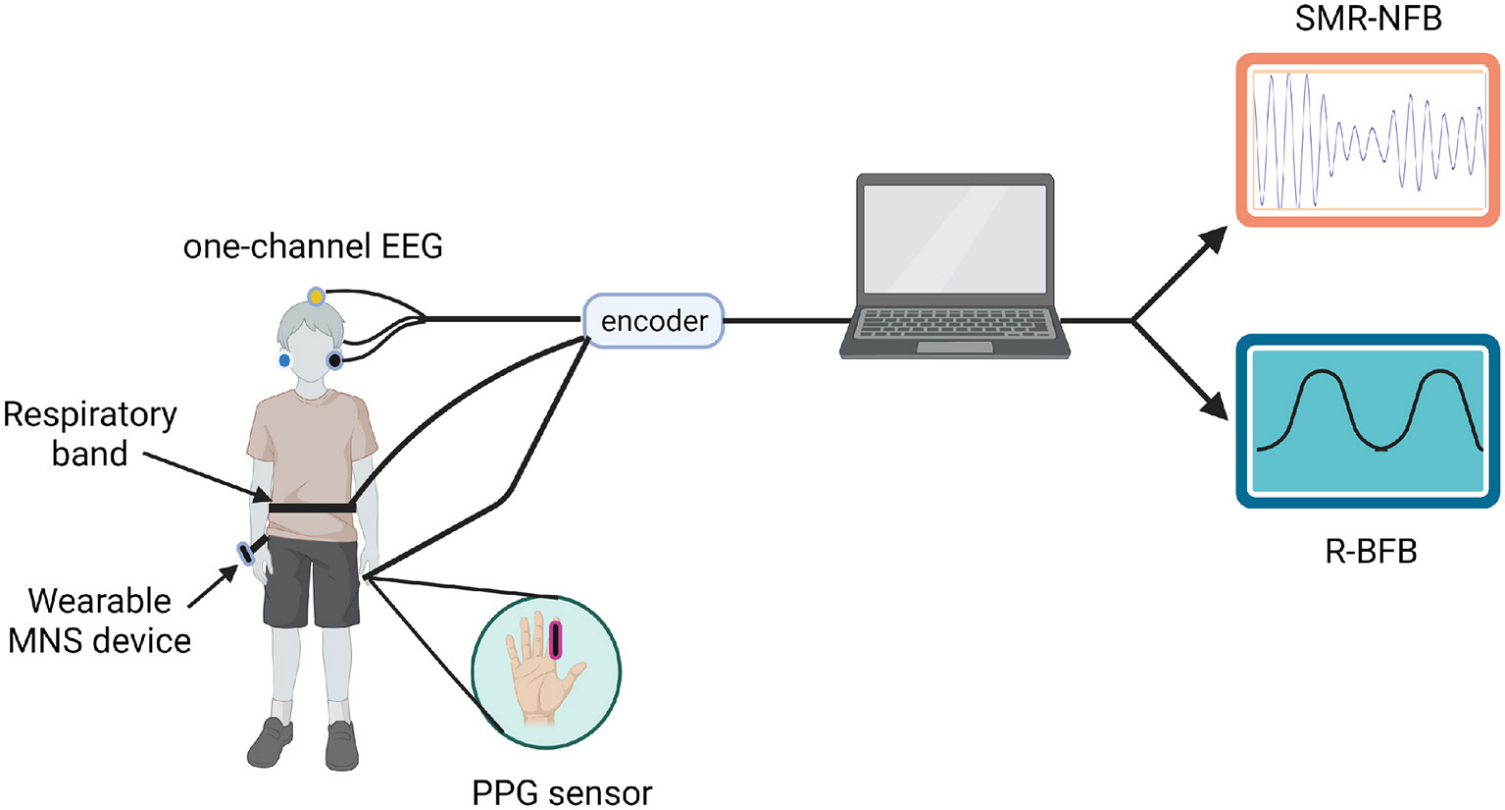
Schematic representation of the proposed therapy. MNS, median nerve stimulation; SMR-NFB, sensorimotor rythm neurofeedback; R-BFB, respiratory biofeedback. Created with BioRender.com.

Participants received a total of 10 sessions through 5 consecutive days per week over a two-week period. This training protocol was based on previous research showing the effects of operant conditioning after SMR-NFB (Morales-Quezada et al., 2019). Each intervention session had two stages (Figure 2): (1) 5 minutes of R-BFB training with MNS; followed by (2) 20 min of SMR-NFB training with MNS. Both groups were exposed to verum R-BFB and SMR-NFB, however, the active group (AG) received active MNS, whereas the sham group (SG) received sham MNS. R-BFB and SMR-NFB were delivered using the ProComp Infiniti Encoder, an 8-channel, battery-powered system for real-time physiological data acquisition (Thought Technology, Montreal).

#### 2.4.1 Respiratory biofeedback

The R-BFB training was designed to induce a state of relaxation. The R-BFB protocol was individualized for each participant after a 5-minute baseline recording of respiratory and pulse rates. A respiration sensor (SA9311M, Thought Technology, Montreal) strapped around the participant's abdomen converted the expansion and contraction of the abdominal area into a signal that rose and fell on the computer screen. After obtaining the mean respiratory and pulse rates, a therapist guided each participant to slow their breathing through deep inhalations, as tolerated. Once the participant learned to breathe in and out following a slow and regular pattern, they engaged in paced breathing following graphic feedback via computer animations and received coaching from the therapist throughout the R-BFB sessions.

#### 2.4.2 Neurofeedback

EEG signals for the NFB sessions were recorded using passive Ag/AgCl electrodes for bipotential measurements. The active electrode was placed at Cz with reference to linked earlobes. Participants were instructed to stay attentive to the task, relax their muscles, and find their own mental strategy to control the “challenges” in the “game.”

Feedback parameters followed these presets: theta (4–7 Hz) inhibition at least 20% below the participant's automatically calculated threshold, SMR (12–15 Hz) reinforcement 80% of the time, and high beta (25–35 Hz) inhibition at least 20% below their threshold. The automatic threshold calculation was based on a moving 30-second window average, adapting to maintaining the required average percentage of success for inhibition/excitation within that period. The threshold could adjust every 2 seconds by 0.2 microvolt increments to maintain an 80% success rate (Morales-Quezada et al., 2019).

SMR-NFB sessions consisted of 5 trials of 3 min each, with inter-trial intervals of 30 seconds, and the whole session lasted approximately 20 min. The visual display for participants included a puzzle with three bars representing each frequency band. One piece of the puzzle was open, and the bars turned green whenever the participant achieved the parameters for 0.5 seconds, indicating a positive reward. This was further reinforced by an auditory stimulus in the form of a bell sound. Additionally, as participants completed subsequent puzzles, they could see a numerical reward of the points earned.

#### 2.4.3 Median nerve stimulation

ADHD patients present a relationship between executive control deficits and motor inhibition (Barkley, 1999). In this population, hypoactivation in fronto-striatal and frontoparietal circuits during inhibitory tasks has been widely described (Hart et al., 2013; Cortese et al., 2012). We hypothesized that MNS could facilitate adequate behavioral responses by modulating thalamo-cortical inhibitory inputs with the electrical stimulation. These circuits engage the sensorimotor system (M1/S1 cortex) serving as a primer for supplementary co-activation of distant networks when a subject is being exposed to cognitive or physical tasks. Moreover, if targeted training is introduced, MNS seems to enhance the effects of such activity by promoting processes associated with neuroplasticity (Houlgreave et al., 2022). Overall, following this rationale, MNS could be used as an adjuvant technique to facilitate inhibitory circuits and enhance attention function in ADHD patients. In this work, the MNS device used was an investigational, battery powered Qey-DTx stimulator (Cinch GmbH, Switzerland). Stimulation electrodes were placed proximal to the right wrist over the anatomical site for the median nerve (approximately 1 cm above the transverse carpal ligament). We applied MNS delivering a maximum of 2 mA of current for the duration of each of the R-BFB (5 minutes) and SMR-NFB (20 min) training sessions. These parameters were selected based on most stimulation characteristics using electrical stimulation with weak currents, and focusing on promoting EEG entrainment (Carvalho et al., 2018). Thus, MNS was delivered at a random frequency between 4–10 Hz during R-BFB to promote a state of relaxation, while a randomly oscillating frequency delivered between 12-16 Hz were used to facilitate SMR entrainment during NFB. For the sham condition, stimulation was applied for a period of 30 seconds and then the device turned off automatically.

### 2.5 Clinical analysis

The data is available upon reasonable request. Requests to access the datasets should be directed to eduardo.santamaría.vazquez@uva.es. Statistical analysis was performed using STATA v.13.1 software (STATA Corp, College Station, TX). The statistical significance level was defined with two-tailed *p*-value < 0.05. Confidence intervals were defined at the 95% confidence level. Descriptive statistics (mean, frequency, range, and percentage) were used to describe socio-demographic variables. We performed univariate analysis using paired *t*-tests applied over the difference between clinical metrics of both groups at baseline, post-intervention and follow-up.

We conducted an exploratory analysis using multiple linear regression with the main study outcome scores from the Conners ADHD rating scale, which includes items on behavioral problems, anxiety, hyperactivity index, learning problems, psychosomatic symptoms, and impulsivity-hyperactivity. We included age, gender, pharmacological treatment status, and type of medication at the time of study participation as covariates. The models were adjusted for each outcome based on pre-established assumptions about effect modification.

### 2.6 EEG analysis

EEG signals of the evaluation sessions were analyzed to find neurophysiological evidence supporting the findings that were reached in the clinical analysis. In the following subsections, we detail the signal processing methods that have been applied for this analysis.

#### 2.6.1 Preprocessing

This stage was aimed to eliminate noisy artifacts from the EEG and increase the signal to noise ratio. We applied a band-pass frequency finite impulse response (FIR) filter of order 1000 between 1 and 70 Hz and a notch FIR filter to eliminate the power line interference in the band between 59 and 61 Hz. The signal was then subsampled to 200 Hz. Afterwards, we split the signal in epochs of 5 seconds, which were used to perform the analysis. It is worthy to mention that we also applied an automatic artifact rejection algorithm to remove noisy epochs (e.g., blinks, jaw contraction, etc.). The algorithm eliminates epochs containing samples with amplitudes exceeding 8 times the standard deviation. This threshold was determined heuristically through visual inspection of the most prevalent artifacts in the dataset, ensuring the effective removal of noise while preserving relevant data.

#### 2.6.2 Spectral analysis

The analysis of the spontaneous brain activity is often approached by measuring the strength of its oscillatory components in different frequency bands using the Power Spectral Density (PSD). In this regard, there is wide evidence that neuropsychiatric disorders affect the normal power distribution of brain rhythms, including ADHD. In this work, we applied spectral analysis to assess possible changes in different biomarkers related to ADHD between baseline, post-treatment and follow-up evaluation sessions to complement the clinical assessment. The PSD of the EEG epochs was estimated with a frequency resolution of 0.5 Hz and 50% overlap using the Welch's method (Welch, 1967). We focused our analysis on Theta [4–8 Hz], Alpha [8–13 Hz] and Beta [13–30 Hz] bands, as these frequency ranges have been extensively linked to ADHD (Snyder and Hall, 2006). In particular, we calculated the Theta-Alpha ratio (TAR) and the Theta-Beta ratio (TBR) to analyze the relationship between these brain rhythms. While TBR has been suggested as a potential biomarker for ADHD diagnosis, a comprehensive review by Arns et al. (2013) raised questions about its reliability. Consequently, we also investigated TAR as an alternative metric, considering the developmental delays often observed in children with ADHD. Additionally, we computed the Spearman rank-order correlation coefficient to explore potential associations between changes in Theta and Alpha power and clinical outcomes.

## 3 Results

### 3.1 Clinical analysis

A total of 60 children with ADHD diagnoses were enrolled and randomized to participate in this trial to receive active MNS and R-BFB/NFB (AG) and sham MNS and R-BFB/NFB (SG). All participants tolerated the interventions well, and no direct side effects associated with any of the treatment arms were reported. Demographics, clinical characteristics, and medications of the population are detailed in Table 1.

**Table 1:** Demographics and baseline assessment.

		**Active group**			**Sham group**		
		**Mean**	**SD**	**N (%)**	**Mean**	**SD**	**N (%)**
Demographics	Age	10.9	2.18		11.44	2.42	
	F			8 (25.8%)			3 (10.4%)
	M			23 (74.1%)			26 (89.6%)
Drugs	Stimulants			6 (19.3%)			6 (20.6%)
	Antidepressants			2 (6.4%)			2 (6.8%)
	Antipsychotics			3 (9.6%)			1 (3.4%)
	Antiepileptics			2 (6.4%)			1 (3.4%)
Conner's baseline scores	Behavior problems	57.54	12.89		58.28	16.22	
	Learning problems	72.06	15.41		71.93	13.20	
	Impulsivity-hyperactivity	67.95	15.46		63.48	13.80	
	Anxiety	60.76	13.95		59.31	11.63	
	Psychosomatic symptoms	52.12	13.35		53.75	14.19	
	Hyperactivity	68.58	12.81		66.82	12.86	

**Figure 3:**
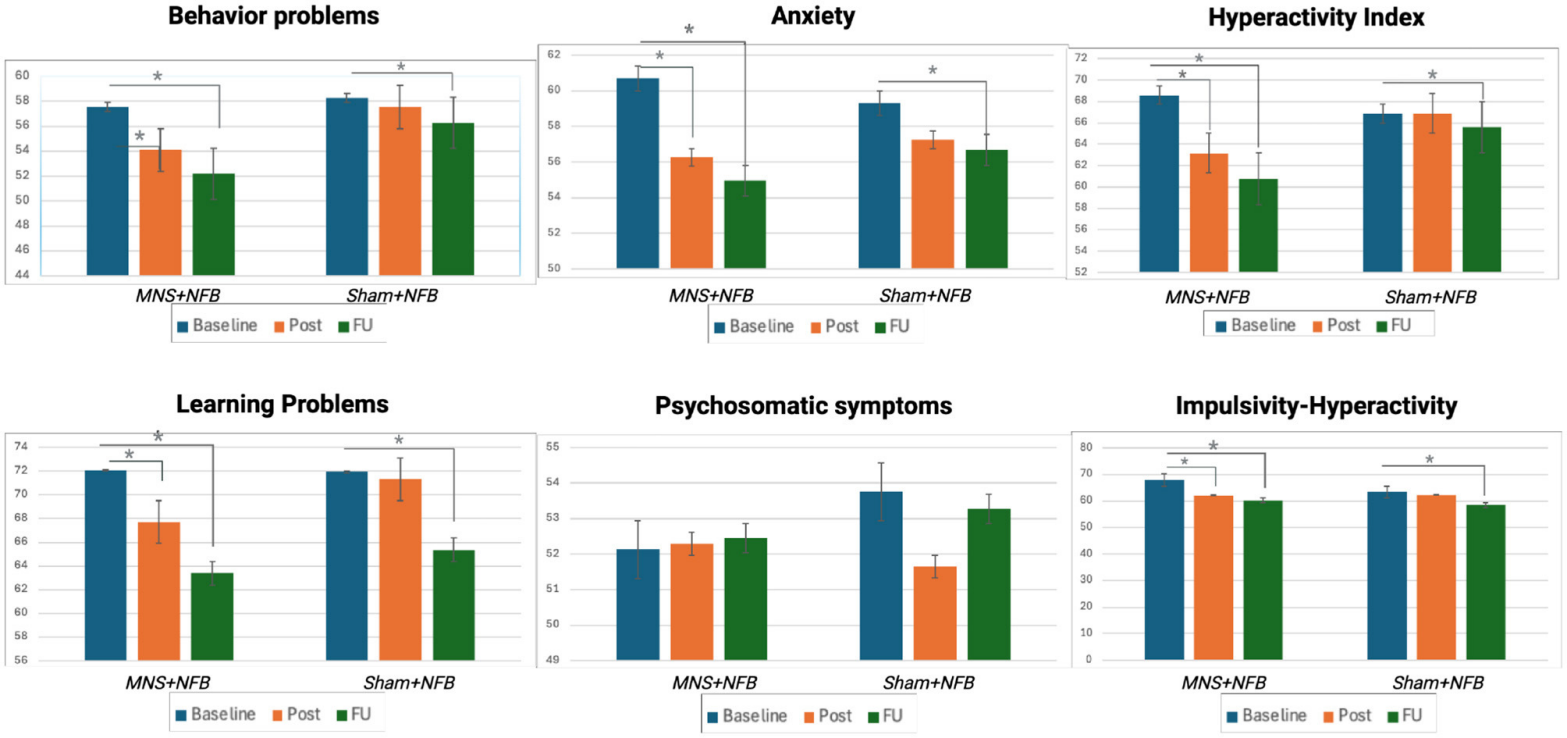
Visual representation of the clinical outcomes across the 3 evaluation time points (Baseline, Post-intervention, and Follow-up) for both active group (AG) and sham group (SG). Bars represent mean scores of Conner's Parent Rating Scale subscales. Significant improvements are indicated by asterisks (*), highlighting reductions in symptoms from baseline to post-intervention and follow-up.

Tables 2, 3 and Figure 3 present the results of paired *t*-tests conducted within the groups to compare post-treatment and follow-up scores with baseline scores. In the AG group, there was a significant improvement observed in several instrument categories, such as behavior problems, anxiety, hyperactivity Index, learning problems, and impulsivity-hyperactivity, both immediately after the intervention and at follow-up. These improvements had small to medium-sized effects. On the other hand, the SG group showed significant symptom improvement only at follow-up, not immediately after the intervention. However, when comparing the AG and SG groups using unpaired *t*-tests, no statistically significant differences were found in any of the Conner's ADHD subcategories. Our results also revealed an elevated standard deviation (SD), which could be expected to the inherent heterogeneity of ADHD presentations. The high SD suggests that responses varied among participants, indicating diverse experiences or behaviors. Additionally, the observed results in the CPRS-R scale may point to varying levels of intervention efficacy across participants. This variability highlights the heterogeneity within the dataset, a factor to consider when interpreting the clinical data.

**Table 2:** Post-treatment vs. baseline.

**Group**	**Variable**	**Mean**	**SD**	***t* stat**.	***p*-value**	**Size effect Cohen's D (Conf. Int)**
**AG**	Behavior problems	−3.45	7.93	−2.42	0.01^*^	−0.29 (−0.79, 0.20)
	Anxiety	−4.45	9.8	−2.5	0.008^*^	-0.34 (−0.84, −0.16)
	Hyperactivity Index	−5.42	11.50	-2.62	0.007^*^	−0.45 (−0.95, 0.05)
	Learning problems	−4.35	13.28	−1.82	0.03^*^	−0.30 (−0.80, 0.19)
	Psychosomatic symptoms	0.16	7.68	0.12	0.55	−
	Impulsivity-hyperactivity	−5.93	13.09	−2.52	0.008^*^	−0.40 (−0.90, 0.10)
**SG**	Behavior problems	−0.72	5.86	−0.67	0.26	−
	Anxiety	−2.06	8.45	−1.31	0.09	−
	Hyperactivity Index	0.034	6.47	0.028	0.51	−
	Learning problems	−0.62	9.71	−0.34	0.36	−
	Psychosomatic symptoms	−2.10	12.98	−0.87	0.20	−
	Impulsivity-hyperactivity	−1.24	9.87	−0.67	0.25	−

The values of each item have been calculated as the value in post treatment minus baseline. Therefore, a negative value implies an improvement in the clinical scales.

^*^Correlation is statistically significant (*p*-value < 0.05).

**Table 3:** Follow-up vs. baseline.

**Group**	**Variable**	**Mean**	**SD**	***t* stat**.	***p*-value**	**Size effect Cohen's D (Conf. Int)**
**AG**	Behavior problems	−4.83	11.37	−2.36	0.01^*^	−0.38 (−0.88, 0.12)
	Anxiety	−5.54	11.59	−2.66	0.006^*^	−0.43 (−0.93, 0.07)
	Hyperactivity Index	−6.64	12.28	−3.01	0.002^*^	−0.50 (−1.00, −0.003)
	Learning problems	−8.67	17.01	−2.83	0.004^*^	−0.62 (−1.13, −0.11)
	Psychosomatic symptoms	−0.41	9.89	0.23	0.59	−
	Impulsivity-hyperactivity	−7.67	14.34	−2.97	0.002^*^	−0.53 (−1.04, −0.02)
**SG**	Behavior problems	−2.82	8.13	−1.87	0.03^*^	−0.18 (−0.69, 0.34)
	Anxiety	−3.24	9.91	−1.75	0.04^*^	−0.29 (−0.81, 0.22)
	Hyperactivity Index	−5.03	10.83	−2.50	0.009^*^	−0.36 (−0.88, 0.15)
	Learning problems	−6.5	10.93	−3.22	0.002^*^	−0.48 (−1.00, 0.03)
	Psychosomatic symptoms	−0.48	11.33	−0.22	0.41	−
	Impulsivity-hyperactivity	−4.96	11.30	−2.36	0.01^*^	−0.36 (−0.88, 0.15)

The values of each item have been calculated as the value in post treatment minus baseline. Therefore, a negative value implies an improvement in the clinical scales.

^*^Correlation is statistically significant (*p*-value < 0.05).

Multiple linear regression models were employed to investigate whether there were differences between groups regarding clinical outcomes assessed with the Conners subscales. Age, gender, and medication were included as covariates in the analysis. However, no significant differences were found between the treatment groups at any time point (see Table 4).

**Table 4:** Linear regression models results.

**Variable**	**Independent variable**	**Coef**.	**Std- error**	**T-ratio**	**Prob**.
Hyperactivity index	Post treatment	−2.78	2.32	−1.20	0.23
	Follow up	−5.86	2.53	−2.53	0.012^*^
	Age	0.43	0.42	1.03	0.303
	Male	5.24	2.48	2.11	0.036^*^
	Medications	−1.00	2.33	−0.43	0.66
	Constant=58.83	Adjust-R2=0.04	F-Ratio=2.50	p=0.03	N=180
Behavior problems	Post treatment	−2.13	2.50	−0.85	0.39
	Follow up	−3.86	2.50	−1.54	0.12
	Age	−0.049	0.45	−0.11	0.92
	Male	8.33	2.68	3.10	0.002^*^
	Medications	−0.44	2.51	−0.18	0.85
	Constant=51.74	Adjust-R2=0.03	F-Ratio=2.43	p=0.03	N=180
Learning problems	Post treatment	−2.55	2.38	−1.07	0.28
	Follow up	−7.65	2.38	−3.22	0.002^*^
	Age	−1.20	0.43	−2.78	0.006^*^
	Male	−1.43	2.56	−0.56	0.57
	Medications	2.92	2.40	1.22	0.22
	Constant=85.99	Adjusted-R2=0.07	F-Ratio=3.97	p < 0.002	N=180

^*^Correlation is statistically significant (*p*-value < 0.05).

After this, we constructed models to evaluate the effect of Conner's subscales without considering the group treatment allocation, at both evaluation time points (after intervention and at follow-up) and the dependent variables of age, gender, and if the participants were taking medication. The hyperactivity index overall regression model was statistically significant (*R*^2^ = 0.06, *F*-value = 2.5, *p*-value = 0.03). It was found that the evaluation at follow-up improved when compared to baseline scores [(β = −5.8], *p*-value = 0.01). Males showed significantly less overall improvement when compared to females [(β = 5.24], *p*-value = 0.03), while age and medication did not significantly predict the hyperactivity index scores at any of the evaluation time points.

The learning problems model was overall statistically significant (R2 = 0.10, F-value = 3.97, *p*-value ≤ 0.002). Both intervention groups significantly improved at follow-up when compared to baseline scores [(β = −7.65], *p*-value = 0.002). Age was also significant, [(β = −1.20], *p*-value = 0.006) indicating that older children improved more than the younger in treatment group.

We did not find statistically significant changes over time for Behavioral problems, anxiety, psychosomatic symptoms, or impulsivity-hyperactivity Conner's subscales.

### 3.2 EEG analysis

**Figure 4:**
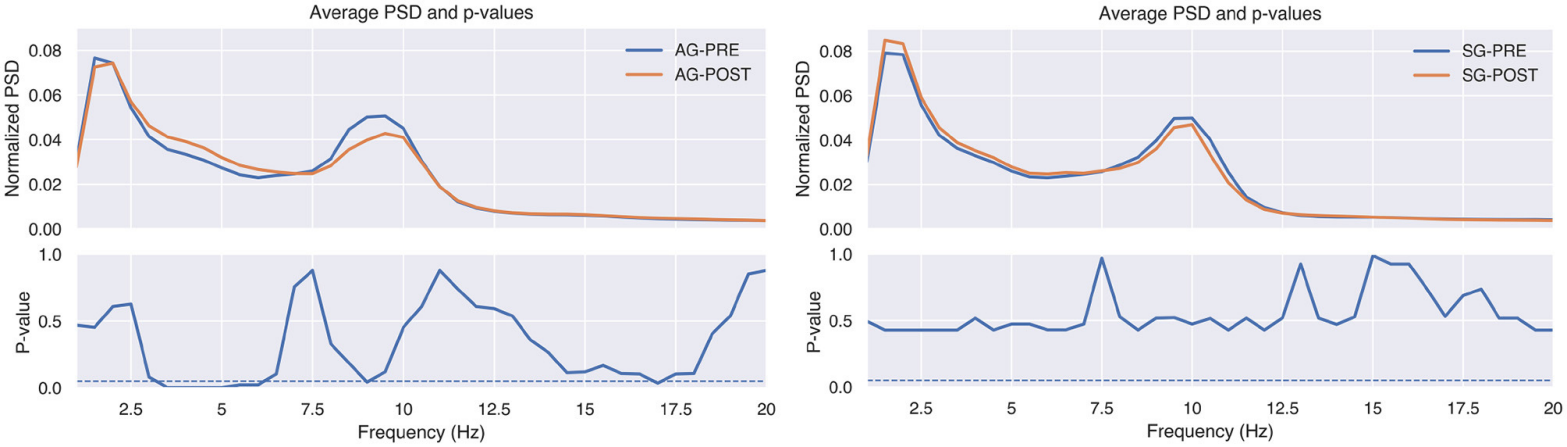
The upper plots show the relative power spectral density (PSD), averaged across patients and EEG channels, for active group (AG) and sham group (SG) in the baseline (PRE) and post-treatment (POST) evaluation sessions. The lower charts show the *p*-values of the comparison at each frequency point, calculated with the Wilcoxon Signed Rank Test and false discovery rate corrected with Benjamini/Hochberg approach. The dashed line indicates the significance level at 0.05.

The EEG analysis also provides some interesting results. Figure 4 shows the averaged PSD of the basal EEG recordings over subjects and channels for AG and SG in the baseline and post-treatment evaluation sessions under the closed-eyes condition. We applied the Wilcoxon Signed Rank Test to find statistical significant changes in PSD values for each frequency point. The false discovery rate (FDR) was corrected following the Benjamini/Hochberg approach (Benjamini and Hochberg, 1995). As can be seen, the power distribution of the AG shifted toward slower frequencies after the intervention. The power of the Theta band increased, reaching statistical significance in the range from 3.5 to 6 Hz (*p*-value < 0.05). On the other hand, the power of Alpha decreased, reaching statistical significance (*p*-value < 0.05) around 9 Hz. These results were not observed in the SG. In this case, no statistically significant changes are detected in the PSD of the EEG between baseline and post-evaluation sessions.

**Figure 5:**
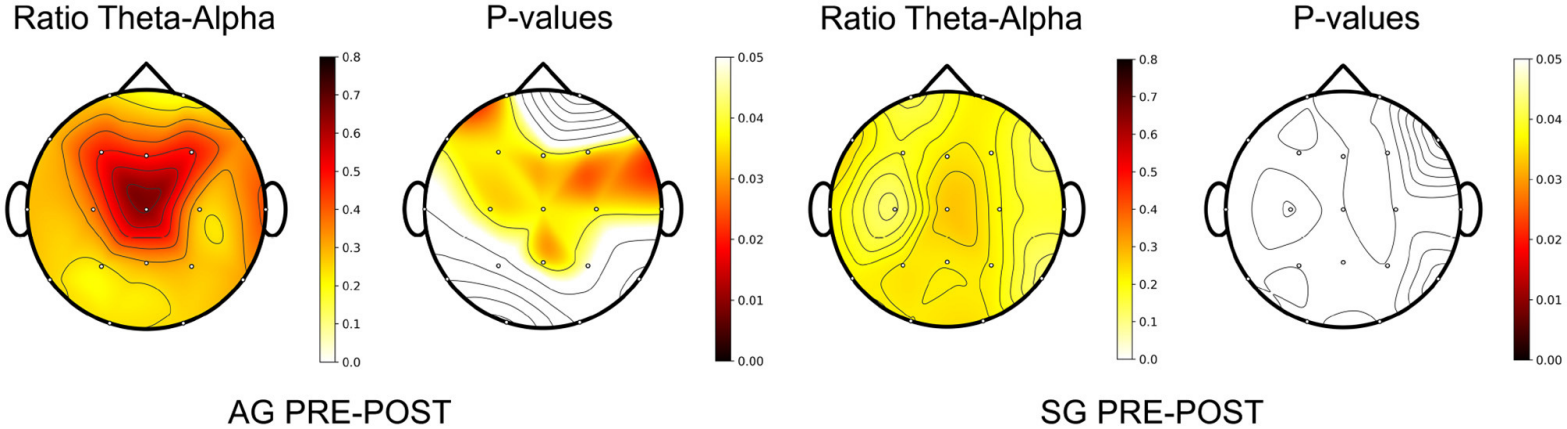
Topography of the Theta-Alpha ratio change between post-tratment and baseline evaluation sessions, averaged across patients, for the active (AG) and sham (SG) groups. *P*-values were calculated with the Wilcoxon Signed Rank Test, correcting the false discovery rate with the Benjamini/Hochberg approach.

Figure 5 shows the scalp distribution of the change between baseline and post-treatment evaluation sessions of the TAR for AG and SG. As before, Wilcoxon Signed Rank Test was applied to calculate the *p*-values, including FDR correction with the Benjamini/Hochberg approach (Benjamini and Hochberg, 1995). As can be observed, the TAR was significantly increased, especially in central electrodes (i.e., F3, FZ, F4, C3, CZ, C4, P3, PZ, P4). In order to study the relationship between Theta and Alpha in these areas with more detail, Table 5 provides the power in these bands averaged across the central electrodes for both groups in pre- and post-evaluation sessions. These results show a statistically significant increase of Theta power while Alpha decreases, confirming the findings of Figure 5. In the case of the TBR, we did not find any statistical differences between groups or between baseline and post-evaluation sessions. Therefore, we did not include the results of this analysis.

**Table 5:** Relative power changes in Theta and Alpha bands between baseline and post-treatment evaluation.

**Group**	**Band**	**Baseline**	**Post**	**Change**	***p*-value**	**Statistic**
AG	Theta	0.2456	0.2667	0.0212	0.0112^*^	85
	Alpha	0.3085	0.2744	–0.0396	0.0345^*^	98
SG	Theta	0.2388	0.2495	0.0107	0.1600	100
	Alpha	0.3037	0.2759	–0.0278	0.0564	83

The statistic is the sum of the ranks of the differences above or below zero, whichever is smaller.

^*^Correlation is statistically significant (*p*-value < 0.05).

**Figure 6:**
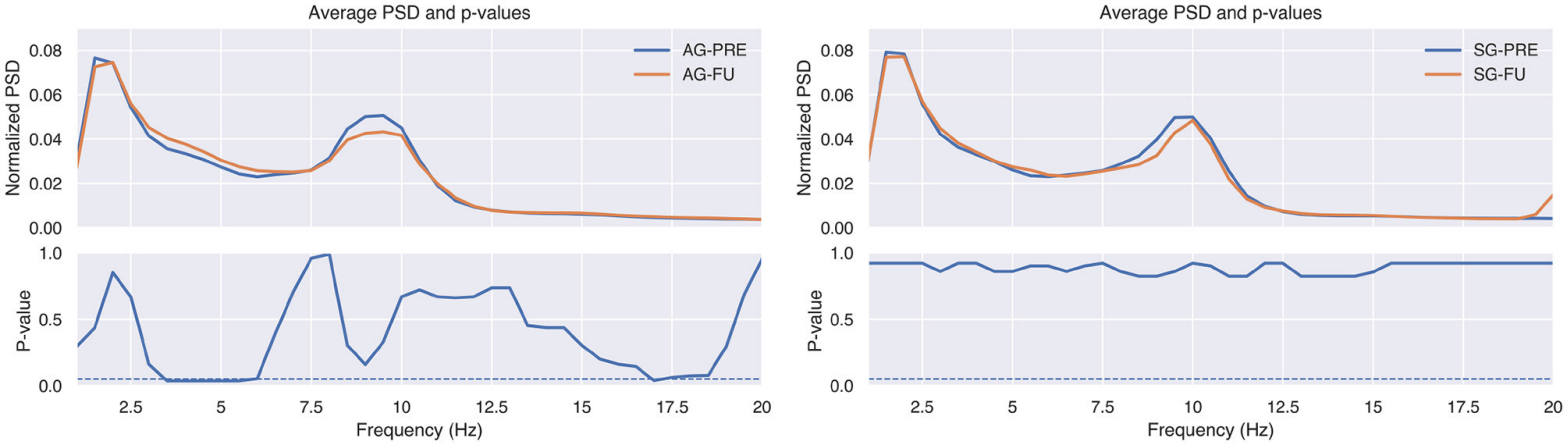
The upper plots show the relative power spectral density (PSD), averaged across patients and EEG channels, for active group (AG) and sham group (SG) in the baseline (PRE) and follow-up (FU) evaluation sessions. The lower charts show the *p*-values of the comparison at each frequency point, calculated with the Wilcoxon Signed Rank Test and false discovery rate corrected with Benjamini/Hochberg approach. The dashed line indicates the significance level at 0.05.

**Figure 7:**
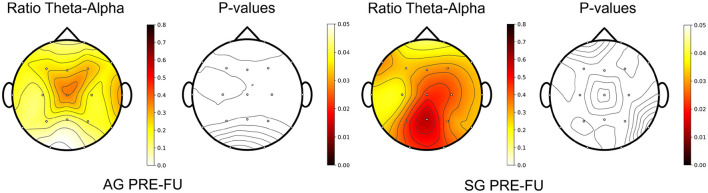
Topography of the Theta-Alpha ratio change between follow-up (FU) and baseline (PRE) evaluation sessions, averaged across patients, for the active (AG) and sham (SG) groups. *P*-values were calculated with the Wilcoxon Signed Rank Test, correcting the false discovery rate with the Benjamini/Hochberg approach.

The same analysis was performed to study the changes between baseline and follow-up sessions. As can be seen in Figures 6, 7, the changes that were appreciated between baseline and post-evaluation sessions are mainly maintained, although their statistical significance has decreased. The same applies to the power in Theta and Alpha bands, which now is only statistically significant for the power increase in Theta, as shown in Table 6.

**Table 6:** Relative power changes in Theta and Alpha band between baseline and follow-up evaluation.

**Group**	**Band**	**Baseline**	**Follow-up**	**Change**	***p*-value**	**Statistic**
AG	Theta	0.2456	0.2628	0.0173	0.0299^*^	99
	Alpha	0.3085	0.2844	−0.0241	0.1775	132
SG	Theta	0.2388	0.2420	0.0031	0.1875	103
	Alpha	0.3037	0.2751	−0.0286	0.2076	105

The statistic is the sum of the ranks of the differences above or below zero, whichever is smaller.

^*^Correlation is statistically significant (*p*-value < 0.05).

Regarding the correlation analysis, Tables 7, 8 show the correlation of the power changes in Theta and Alpha bands with the clinical changes for each variable in AG and SG, calculated with Pearson's method. The *p*-values were calculated with a hypothesis test whose null hypothesis is that the two sets of input data were uncorrelated. For this analysis, we only used the central channels (i.e., F3, FZ, F4, C3, CZ, C4, P3, PZ, and P4) for two reasons: the subjects received MNS and SMR-NFB in this area, and it is where the changes of TAR were more strong (see Figures 5, 7).

**Table 7:** Pearson correlation coefficient (ρ) between EEG biomarkers and clinical outcomes, comparing post-treatment measurements with baseline.

**Grp**	**Band**	**Behavioral problems**		**Anxiety**		**Hyperactivity index**		**Learning problems**		**Psychosomatic symptoms**		**Impulsivity hyperactivity**	
		ρ	*p*-value	ρ	*p*-value	ρ	*p*-value	ρ	*p*-value	ρ	*p*-value	ρ	*p*-value
AG	Theta	0.215	0.282	0.074	0.714	−0.064	0.753	−0.162	0.419	0.266	0.180	−0.303	0.124
	Alpha	−0.044	0.827	−0.033	0.870	0.024	0.905	0.055	0.787	−0.285	0.150	0.081	0.687
SG	Theta	−0.086	0.690	0.030	0.889	−0.333	0.112	−0.600	0.002^*^	0.006	0.978	−0.282	0.182
	Alpha	0.110	0.609	−0.002	0.993	0.344	0.010	0.413	0.045^*^	−0.3380	0.106	0.190	0.374

^*^Correlation is statistically significant (*p*-value < 0.05).

**Table 8:** Pearson correlation coefficient (ρ) between EEG biomarkers and clinical outcomes, comparing follow-up measurements with baseline.

**Grp**	**Band**	**Behavioral problems**		**Anxiety**		**Hyperactivity index**		**Learning problems**		**Psychosomatic symptoms**		**Impulsivity hyperactivity**	
		ρ	*p*-value	ρ	*p*-value	ρ	*p*-value	ρ	*p*-value	ρ	*p*-value	ρ	*p*-value
AG	Theta	−0.389	0.045^*^	−0.0834	0.679	−0.1597	0.426	−0.167	0.406	−0.241	0.226	−0.150	0.456
	Alpha	0.389	0.045^*^	−0.083	0.680	0.145	0.471	0.084	0.679	0.330	0.093	0.251	0.207
SG	Theta	0.130	0.544	0.161	0.453	−0.024	0.910	−0.018	0.934	0.080	0.712	0.034	0.873
	Alpha	−0.3955	0.056	−0.059	0.784	−0.154	0.473	−0.138	0.521	−0.521	0.009^*^	−0.052	0.808

^*^Correlation is statistically significant (*p*-value < 0.05).

## 4 Discussion

This work investigated a non-pharmacological intervention in 60 ADHD children to answer two research questions: whether the proposed R-BFB/NFB intervention could potentially treat some of the symptoms of this neuropsychiatric disorder, and whether the application of MNS could enhance the effects of this protocol in the population under study.

Regarding participants' interaction with R-BFB/NFB paradigms, all participants tolerated the intervention, followed the instructions, and engaged in the tasks. For the R-BFB, participants decreased their respiratory rate at rest to three to four breaths from baseline, indicating adequate following of the audio-visual paced feedback. Similarly, children sustained attention during the NFB entrainment and modulated their endogenous cortical oscillations following the pre-defined parameters of the task. In terms of clinical outcomes, children in the AG demonstrated significant improvements from baseline to the post-treatment evaluation in behavioral problems, anxiety, hyperactivity index, and impulsivity-hyperactivity. In contrast, the SG group did not exhibit any statistically significant changes by the end of the intervention. At the follow-up evaluation, however, both groups showed significant improvements across all Conner's subscales, with the exception of psychosomatic symptoms. While the Conner's Parent Rating Scale relies on parents' subjective perceptions and interpretations of their child's behaviors, making it susceptible to personal, cultural, and situational influences, the results still indicate a notable trend. The AG demonstrated greater mean improvements across most categories compared to the SG, as evidenced by higher effect sizes in favor of the AG. These findings suggest that MNS had a boosting effect on R-BFB/NFB therapy is more effective in treating ADHD symptoms compared to the R-BFB/NFB protocol without stimulation. For the SG group, which received sham stimulation, the follow-up results highlight the significant impact of treatment expectations linked to technology-based interventions, as well as the placebo-by-proxy effect on clinical outcomes (Grelotti and Kaptchuk, 2011). This type of placebo response is important to consider, especially when the absence of loss to follow-up suggests that participants and their caregivers may have developed positive expectations toward the experimental intervention and formed attachments to the staff involved in the experiment (Morales-Quezada et al., 2019). This connection could be attributed to the consistent presence of study staff throughout all training sessions. Moreover, clinical research contexts integrate diverse psychological elements, including learned associations between cues (possibly potentiated by the operant conditioning itself) and past positive experiences. Additionally, conceptual knowledge based on verbal suggestions induces expectations about the intervention and its outcomes. Similarly, social interactions among the participants, their caregivers, and the study staff might mimic the patient-care provider relationship (Wager and Atlas, 2015), which in this case, may triggered the placebo responses.

The results of the EEG analyses support these findings with objective biomarkers, revealing significant alterations in spontaneous brain activity after the intervention for the AG, but not for the SG. Particularly noteworthy is the significant increase in Theta power in the frontal lobe observed in the AG. Theta waves are widely recognized in the literature as being associated with deep relaxation, meditative states, and light sleep (Suetsugi et al., 2000). In this context, reduced respiratory rates—achieved through practices such as mindfulness meditation or paced breathing exercises, as in the case of R-BFB—are known to enhance Theta wave activity (Baijal and Srinivasan, 2010). This shift in EEG activity toward slower frequencies could be attributed to the applied intervention and may explain the observed clinical improvements in behavioral problems, anxiety, and impulsivity-hyperactivity indices in the AG (Aftanas and Golocheikine, 2001). Additionally, it is worth to note the lack of changes observed on the predefined SMR frequency band, despite having a NFB and MNS protocols specifically designed to increase the power and amplitude of this rhythm. On one hand, developmental differences in EEG band frequencies may result in overlaps between SMR and alpha bands in children. This could explain the significant changes observed in the TAR, which may better capture intervention effects in this age group. On the other hand, SMR indicates a cortical idling state or inhibition of the activation of the sensorimotor cortex (Gaetz et al., 2010). Moreover, SMR is elicited in situations where subjects withhold or control the execution of a response, being obtained over sites that probably are under, or exert top-down control (Klimesch et al., 2007). The generation of SMR in the sensorimotor cortex is elicited by stimulating radiations from the nucleus ventralis posteriolateralis of the thalamus (Fairchild and Sterman, 1974), indicating its inhibitory nature over cortico-cortical circuits. It can be argued that, in the setting of this experiment, SMR could have been suppressed by the increase of the thalamo-cortical circuits generating Theta, secondary to the induced relaxation state offered by R-BFB and the Theta-like entrainment triggered by MNS. In this regard, none of the clinical scales improved in the SG, which did not show differences in Theta power with respect to the baseline. Nevertheless, as shown in Table 7, the correlation analysis failed to find significant associations between changes in Theta power and the clinical outcomes, probably due to the limited size of each group and high variability in results in the post-evaluation session. With respect to the decrease in Alpha power, we hypothesize that it is the result of compensatory mechanisms to maintain the brain's homeostasis accounting for the increase in Theta power. Interestingly, the EEG analysis of the follow-up session revealed that some of the physiological changes were maintained one month after the intervention, with the increase in Theta power still showing statistical significance. This suggests that this long-term modification of the Theta rhythm represents a sustained neuroplastic phenomena that may mediate improvements in the AG by promoting more relaxed brain states. In this case, we found a significant negative correlation with the behavioral problems index, associating the increase in Theta power to symptom relief in the questionnaires. As can be seen in Table 8, the rest of correlations are also negative, although none of them reached the statistical significant threshold.

These findings suggest that the proposed R-BFB/NFB protocol may be an effective non-pharmacological therapy for ADHD. Nevertheless, there are clear differences between AG and SG that are worth discussing. The AG, which received verum MNS, improved more items in both post- and follow-up evaluations than the SG, who received sham MNS. Compared to neurotypical populations, ADHD patients show structural and functional alterations in the somatosensory cortex (Duerden et al., 2012). In fact, some of the symptoms that characterize ADHD may be explained by a hyper-excitability of the primary somatosensory area, which leads to an imbalance between excitation/inhibition states (Miyazaki et al., 2007). In this regard, rhythmic MNS has proved to increase synchronism of neural activity in the contralateral somatosensory cortex through afferent pathways, which could potentially lead to enhanced inhibitory responses in this area (Houlgreave et al., 2022). Several studies achieved promising results using MNS in disorders associated with hyperexcitability of sensorimotor cortices, such as chronic tic disorder (Houlgreave et al., 2022; Maiquez et al., 2023). In this study, MNS stimulation modulated the activity of the Theta band at the cortical level of the AG, showing that this technique can have a direct impact on the brain rhythms. Given that the AG performed consistently better in the clinical analysis, MNS may enhance the proposed R-BFB/NFB protocol to decrease ADHD symptoms.

Despite the positive results presented in this paper, we have to acknowledge some limitations that should be considered. This study tested the proposed therapy in 60 subjects, one of the largest samples among related studies of non-pharmacological therapies for ADHD (Enriquez-Geppert et al., 2017). However, due to the large variability of ADHD disorder, a larger sample would be beneficial to increase the confidence of some findings, especially the correlations between EEG biomarkers and clinical results. More important, is that our results may reflect the underpower characteristics of the study design, which prevented us from observing a statistical significance between the groups. Therefore, we propose to increase the sample size in future studies. Another aspect is the difficulty and subjectivity of ADHD diagnosis and assessment, which has been the subject of discussion in the research community (Hinshaw, 2018). To minimize this intrinsic limitation of ADHD research, we followed two of the most accepted criteria: the DSM-5 criteria for ADHD diagnosis, and the CPRS-R for clinical assessment. Moreover, we complemented the assessment with EEG recordings of the brain's spontaneous activity, which provided interesting biomarkers to support the clinical outcomes. Nevertheless, additional analyses such as online NFB performance to evaluate individual's ability to self-regulate the targeted EEG frequencies through training sessions, or complex connectivity-based and graph theory methods, might complement these findings with new perspectives, representing an interesting future line of research. It is important to note that a subset of participants (41.7% in the AG and 34.2% in the SG) were undergoing pharmacological treatment during the study, which may have influenced the results. While our statistical analysis indicated no significant impact of medication on the clinical outcomes, pharmacological effects on EEG metrics (e.g., TBR/TAR) cannot be disregarded. For example, stimulants can modulate EEG by increasing beta activity and reducing alpha oscillations, which reflects an enhanced state of arousal. To more accurately isolate the intervention effects, future studies should ideally be conducted with pharmacologically-naïve participants. Another characteristic from our sample was the higher proportion of males in each arm when compared to females. We acknowledge that our sample was unbalanced for sex, although this imbalance is expected due to ADHD epidemiological data where boys are three to five times more affected than girls.

## 5 Conclusions

This study aimed to assess the efficacy of a non-pharmacological intervention utilizing R-BFB/NFB and MNS in alleviating ADHD symptoms among 60 children. The findings revealed noteworthy enhancements in behavioral issues, anxiety levels, hyperactivity, and learning difficulties compared to baseline measures. Moreover, the positive outcomes observed in the AG receiving verum MNS were notably stronger than those in the SG group, who received sham MNS. EEG analysis further supported these results by demonstrating significant alterations in spontaneous brain activity post-intervention. Specifically, an increase in Theta power within the frontal lobe, observed in the AG, appeared to correlate with improvements in behavioral problems, anxiety levels, and impulsivity-hyperactivity indices. These improvements were sustained during the follow-up session, indicating the potential effectiveness of the proposed protocol as a non-pharmacological therapy for ADHD.

## Data Availability

The raw data supporting the conclusions of this article will be made available by the authors, without undue reservation.
